# Use of Off-Label Drugs and Nutrition Supplements among Patients with Amyotrophic Lateral Sclerosis in Norway

**DOI:** 10.1155/2022/1789946

**Published:** 2022-04-12

**Authors:** Gard Aasmund Skulstad Johanson, Ole-Bjørn Tysnes, Tale L. Bjerknes

**Affiliations:** ^1^Department of Clinical Medicine, University of Bergen, Bergen, Norway; ^2^Neuro-SysMed, Department of Neurology, Haukeland University Hospital, Bergen, Norway

## Abstract

**Materials and Methods:**

A cross-sectional questionnaire study was performed, where 41 ALS patients reported their use of off-label treatments, as well as self-perceived HRQOL using the RAND-12 questionnaire.

**Results:**

A majority of respondents used riluzole. Of the 41 respondents, 18 (43.9%) reported use of off-label medications and 18 (43.9%) used nutritional supplements. Low-dose naltrexone was the most commonly used off-label medication, whereas vitamins accounted for most of the nutritional supplements. The respondents' RAND-12 component scores were significantly lower than those of the general population. Low-dose naltrexone and vitamin B were associated with a better physical component score.

**Conclusions:**

Most of the respondents in our study adhere to the recommended treatment protocols, as less than half of them reported using off-label medications or nutritional supplements against ALS. Positive correlations between physical HRQOL and use of low-dose naltrexone or vitamin B were demonstrated. These results warrant further investigations.

## 1. Introduction

Amyotrophic lateral sclerosis (ALS) is a severe neurodegenerative disease characterized by gradual paralysis and muscle atrophy due to combined upper and lower motor neuron dysfunction in the brain, brainstem, and spinal cord [1, 2]. The incidence of ALS is 0.6–3.8 per 100000 person years [3]. Studies from northern Norway suggest the incidence in Norway is 2.1 per 100000 person years [4]. The disease is progressive, with death resulting from respiratory failure 24–50 months from the time of diagnosis [1, 3, 4]. Although recent studies have identified several genes and cellular pathways involved in the pathogenesis of ALS, the causes of the disease are still largely unknown [1, 5].

The only approved treatment for ALS in the EU and Norway is riluzole, which extends mean survival by 3–6 months [5–8]. Other available treatments focus on symptomatic management and respiratory support. Recent studies have shown that multidisciplinary follow-up increases quality-of-life (QoL) in ALS patients [9] and can also prolong survival [10, 11]. Edaravone, a drug limiting cellular stress by reducing production of reactive oxygen species (ROS), is approved in the United States and Japan, but has only a modest effect in delaying motor symptoms in ALS [12].

Off-label use of medications is usually described as any use of a drug outside its approved indications [13]. In several serious diseases, such as cancer, Alzheimer's disease, epilepsy, and frontotemporal dementia, off-label use of medications is common [14–16]. In a study of 120 patients with Parkinson's disease from the USA, 63% reported using nutritional supplements [17], although the evidence to support clinical efficacy of nutritional supplements is inconclusive [18].

Although there are data suggesting positive effects of several substances on the clinical course of ALS, riluzole and edaravone remain the only FDA approved drugs against this disease [19–21]. Patient Internet forums and websites, such as “Patients like me” [22], suggest possible off-label medications against ALS. In addition, “ALS untangled” is an initiative that systematically reviews alternative and off-label treatments for ALS, enabling patients and clinicians to make more informed choices [23, 24]. For example, vitamin B, metformin, and low-dose naltrexone have been discussed as possible future treatments against ALS [25–27], but the effects of these medications on ALS are not thoroughly studied in humans. To the best of our knowledge, studies on ALS patients' use of nutritional supplements and off-label medications are scarce [28, 29].

The aim of this study was to describe the use of off-label medications and nutritional supplements among ALS patients in Norway and investigate whether the use of such treatments is related to changes in self-perceived physical or mental health.

## 2. Materials and Methods

### 2.1. Design

An anonymous, cross-sectional, questionnaire study was performed, asking about use of off-label treatments and self-perceived health status. The RAND-12 Health Status Inventory (RAND-12) was used to measure health-related quality-of-life (HRQOL) [30–32].

### 2.2. Study Participation and Recruitment

ALS patients with or without frontotemporal dementia and age above 18 years were included in the study.

The questionnaire was distributed to patients through the two ALS patient organizations in Norway, as well as the website of the study centre (Neuro-SysMed at Haukeland University Hospital, Norway). In addition, clinicians at neurological departments in Norwegian hospitals were asked to give their ALS patients an invitation to the study. Data were collected between February 15th and May 1st, 2021.

### 2.3. Instruments

The questionnaire was separated into two parts. The first part contained 14 items on general demographic and clinical information and use of off-label drugs and nutritional supplements. The questionnaire combined predefined alternatives and space for the respondents to give information as free text. The background information items included age, sex, educational level, time since diagnosis, and aspects of the respondents' medical follow-up. This was followed by items regarding the use of riluzole and off-label medications, prescribing health personnel, where the respondents sourced information on the drug(s), use of supplements, and an item on where the respondents had gained information on the supplement(s). The questions concerning respondents' medical follow-up, off-label drug use, prescribing health personnel, information sources on off-label drugs, use of nutritional supplements, and info on nutritional supplements were multiple choice questions. Part one of the questionnaire was developed by the authors based on clinical experience and review of literature on relevant off-label medications and supplements.

The second part included the RAND-12 questionnaire, provided in Norwegian by the centre on patient-reported outcomes data [30, 33]. The RAND-12 questionnaire contains the same questions as the medical outcomes study short form health survey-12 (SF-12), but they differ in regards to the scoring algorithm [30–32].

The RAND-12 comprised of twelve items, in eight scales. The eight scales are (with items per scale in parenthesis) as follows: physical functioning (2), role physical (2), bodily pain (1), general health (1), vitality (1), social functioning (1), role emotional (2), and mental health (2) [31, 34, 35]. The RAND-12 produces two component scores based on the twelve items, the physical component score (PCS12) and mental component score (MCS12). The scoring algorithm uses all twelve items in calculating both PCS12 and MCS12. The algorithm assumes a correlation between MCS12 and PCS12 [31]. RAND-12 population data from a general Norwegian population (*n* = 4987) was provided upon request by the centre on patient-reported outcomes data [36]. Responses from patients were collected anonymously using SurveyXact.

### 2.4. Data Analysis and Presentation

The respondents' use of off-label medications and nutritional supplements were given in frequencies and percentages. RAND-12 component scores were calculated according to guidelines provided by the centre on patient-reported outcomes data [30]. To facilitate comparative analyses, the general population data were grouped in similar age and sex categories as the patient-reported answers in the questionnaire. Each respondent was assigned an expected physical and mental component score, equal to the mean component scores of their age and sex category in the general population norms. A paired sample *t*-test was used to test the difference in HRQOL between the respondents and the general population. Correlations between off-label medications, use of nutritional supplements, and PCS12 or MCS12 were calculated using two-tailed Spearman correlation. SPSS (version 26.0.0.0) was used for data management and analysis.

### 2.5. Ethics

Before conducting the study, the Regional Committee for Medical and Health Research Ethics West was consulted and found that the study did not require ethical approval, as long as responses were collected anonymously. The data protection officer at The University of Bergen (UoB) gave further advice on measures to ensure anonymity. As recommended by the data protection officer, the study was registered in the system for risk and compliance at UoB.

## 3. Results

### 3.1. Patient Population

A total of 41 respondents answered part one of the questionnaire, concerning medications, off-label drug use, and nutritional supplements. Of these, 36 completed the entire questionnaire including the RAND-12 questions. The descriptive statistics include all 41 respondents, whereas correlations and RAND-12 component scores only include the 36 respondents that finished both parts one and two.

Most of the respondents reported having been diagnosed with ALS in the last two years (53.7%). Nine (22%) reported a time since diagnosis of more than five years.

### 3.2. Off-Label Medications

A total of 18 (43.9%) respondents reported using off-label medications of any kind against ALS (Table 1), whereas 23 (56.1%) used riluzole in monotherapy. Low-dose naltrexone was the most common off-label medication used by eight respondents (19.5%). In addition, seven (17.7%) respondents reported being part of the NO-ALS study, a randomized placebo-controlled clinical intervention study of nicotinamide riboside/pterostilbene supplement in early ALS (clinicalTrials.gov ID: NCT04562831) (Table 1). None of the respondents reported using edaravone. Nine patients reported use of medications against anxiety, depression, or sleeping problems (not shown).

### 3.3. Nutritional Supplements

A total of 18 (43.9%) of respondents reported using nutritional supplements specifically against ALS (Table 2). Vitamins D and B were most common, with nine (22.0%) and eight (19.5%) users, respectively.

### 3.4. Information Sources

Neurologists were the most common information source on off-label medications, reported by 12 (29.3%) of the respondents. The second most common information source was the Internet, 7 (17.1%) respondents, followed by patient organizations and forums, 5 (12.2%) respondents. Patients could also choose the following categories: “general practitioner” (*n* = 3), “other healthcare provider” (*n* = 2), and “others” (*n* = 2). When limiting only to those who reported using off-label drugs (*n* = 18), neurologists and the Internet was equally common, each with six (33.3%) listing these as their information source.

Concerning nutritional supplements, the Internet was the most common information source reported by 10 (24,4%) respondents. Nine (21,9%) reported that they had sourced information on nutritional supplements against ALS from patient associations, forums, shops selling nutritional supplements, pharmacies, or alternative treatment providers.

### 3.5. RAND-12

The responding ALS patients had significantly lower RAND-12 component scores than the general Norwegian population (Table 3). The mean PCS12 in the respondent group was 33.8, compared to 51.8 in the general population (*p* < 0.001), and mean MCS12 was 39.7, compared to 51.8 in the general population (*p* < 0.001) (Table 3).

When analysing individual RAND-12 items, the responses concerning physical limitations were generally poor, whereas responses to the items on mental health limitations were better. When asked if their physical health had limited them during the last four weeks, thirty-four respondents (94.4%) answered “yes,” while two (5.6%) answered “no.” When asked the same question with regards to mental health, twenty-one (58.3%) answered “yes” and fifteen (41.7%) answered “no.”

Furthermore, 29 respondents (80.1%) reported having felt down or depressed “some of the time” or less. On the other hand, about half of the respondents (47.2%) reported having had a surplus of energy “some of the time” or more the last four weeks. When asked about how much of the time respondents had felt “calm and relaxed,” 26 (72,2%) reported having felt as such “some of the time” or more. Only two (5.6%) respondents reported never having felt calm or relaxed during the last four weeks.

### 3.6. Correlations

Comparing MCS12 and PCS12 with the use of off-label medications or supplements, we found significant positive correlations between PCS12 and use of low-dose naltrexone (0.495, *p* < 0.001) and between PCS12 and use of vitamin B supplements (0.444, *p* < 0.001).

No correlations were found between MCS12 and use of either vitamin B or low-dose naltrexone and sex or age categories.

## 4. Discussion

The main finding of the present study is that although more than half of the respondents adhere to the approved treatment protocol with riluzole in monotherapy (56,1%), 18 (43.9%) respondents reported using off-label medications. This prevalence is somewhat higher than that reported in a study of US ALS patients [29]. Moreover, 18 of our responding ALS patients (43,9%) reported using nutritional supplements. This is comparable to findings in a German study [28], but lower than the prevalence reported in US ALS patients [29]. Given the lack of new treatment options against ALS and use of off-label medications by patients in other severe diseases [13–15], it is not surprising that off-label medications and nutritional supplements are used among ALS patients.

In our study, low-dose naltrexone was the most commonly used off-label drug, and using low-dose naltrexone was associated with significantly higher PCS12. There is no set definition, but naltrexone in doses from 3 to 4.5 mg is typically considered as low dose [26, 37]. Low-dose naltrexone is hypothesized to influence immunomodulation, and a neuroprotective effect of the similar drug naloxone has been mentioned when discussing low-dose naltrexone against ALS [26]. Evidence to support these effects are lacking at this time [26]. The low number of respondents in our study suggests that the association between the use of low-dose naltrexone and PCS12 will need verification in future studies. Naltrexone in normal doses is associated with side effects such as liver toxicity, but side effects should be limited considering the low doses administered in low-dose naltrexone [26, 38].

Seven of the 18 respondents using off-label medications reported being participants in the NO-ALS study. These respondents use off-label treatment or placebo organized by healthcare personnel in their local ALS clinic. As part of the clinical trial, patients are asked to refrain from using vitamin B3 supplements and blueberry concentrates (clinicalTrials.gov ID: NCT04562831). Participating in this study will influence these respondents' use of some off-label treatments directly and may also result in these respondents being more restrictive in trying off-label drugs and nutritional supplements in general.

Vitamin D was the most used nutritional supplement, but no significant correlation between vitamin D and HRQOL was found. Although some have suggested vitamin D as a possible treatment against ALS, this has not been supported by the scientific studies published so far [39–43].

Vitamin B was the second most used nutritional supplement, and the use of vitamin B was associated with significantly higher PCS12. Due to the limitations in our study, it is not possible to know whether this correlation is due to a cause-effect relationship or other factors. Some studies have found promising effects of different forms of vitamin B on ALS [44, 45]. A recent phase II/III RCT indicated that ultra-high dose methylcobalamin (vitamin B12) had a positive effect on disease progression in a subset of ALS patients [45]. However, at present, there is not sufficient evidence to recommend any B vitamins specifically against ALS [27, 44, 45]. Moreover, in other neurodegenerative diseases, studies on the efficacy of vitamin B as a treatment option in patients without an underlying deficiency are also conflicting [46–48].

None of the respondents reported using edaravone, which is approved for treatment of ALS in the USA and Japan. Treatment with edaravone is expensive, and this may be a deterrent against using this drug off-label [49].

It is worth noticing that more than half of the respondents in our study use riluzole in monotherapy and that seven respondents used off-label treatment as part of the NO-ALS clinical trial. These numbers mean that most respondents adhere to clinicians' recommended treatment protocols and may suggest that patients trust that the ALS clinics are offering the best available treatment.

Off-label treatments without a documented effect can be a challenge to patients and healthcare providers. Clinicians caring for ALS patients are regularly consulted about possible off-label treatments, but the safety and efficacy of the drugs are often unknown, and the treatments may be costly. There are, however, some resources to guide patients and clinicians on off-label treatments against ALS, such as the ALS untangled initiative [23, 24].

Compared to a Norwegian general population, the respondents in our study had significantly lower RAND-12 mental and physical component scores. Mean PCS12 in our study is comparable to studies measuring HRQOL in ALS patients using the short form-12 (SF-12) [50]. Mean MCS12 was higher than mean PCS12, but was somewhat lower than previously reported SF-12 MCS data [50]. A possible explanation for this is that the RAND-12 scoring algorithm, unlike the SF-12 algorithm, assumes a correlation between PCS12 and MCS12 [31]. Moreover, differences between the responding populations may also have contributed to the small divergence. Furthermore, ALS patients may have a stable self-reported general QoL even as their disease progresses [1, 2, 51]. QoL is influenced not only by physical functioning but also encompasses social, spiritual, and existential factors [51, 52]. Findings supporting that many ALS patients maintain general QoL even as physical function deteriorates should be taken into consideration when planning treatment and follow-up of this patient group.

### 4.1. Study Strengths and Limitations

The present study is one of the first describing the use of off-label treatments in ALS. Knowledge on patients' complete use of supplements may be useful for clinicians planning symptomatic treatment, for instance to avoid drug interactions. Clinicians may also give advice on the safety aspects of supplements, as these are not subjects to the same controls as approved medications.

Our study is limited by its size, and as the respondents are anonymous with no randomisation and the study is relying on self-reported data and nonsystematic recruitment, the study is vulnerable to biases.

## 5. Conclusions

In this study, we have found that most responding ALS patients adhere to the recommended treatment protocols and that less than half of the responding ALS patients used off-label drugs or nutritional supplements against ALS. In line with results from earlier studies, the respondents' HRQOL was lower than the general population norm. Our findings may suggest a positive correlation between the ALS patients' physical HRQOL and use of low-dose naltrexone or vitamin B. The findings in this study warrant further investigation.

## Figures and Tables

**Table 1:** Patients reporting off-label use of medications against ALS.

Medication	*n* (%)^†^
Low-dose naltrexone	8 (19.5)
Drug studies (NO-ALS)^‡,§^	7 (17.7)
Dextromethorphan hydrobromide/quinidine sulfate (Nudexta)	2 (4.9)
Ropinirol	1 (2.4)
Metformin	1 (2.4)

^†^Each respondent could report more than one medication; either by choosing predefined alternatives or give information as free text. ^‡^The NO-ALS study is a randomized placebo-controlled clinical intervention study of nicotinamide riboside/pterostilbene as a supplement in early ALS (clinicalTrials.gov ID: NCT04562831). ^§^The NO-ALS study was the only study that respondents reported being part of and the only available drug study in Norway. It is not known whether respondents are in the intervention or placebo group.

**Table 2:** Patients reporting use of nutritional supplements against ALS.

Name of nutritional supplements	*n* (%)^†^
Vitamin D	9 (22.0)
Vitamin B	8 (19.5)
Vitamin E	3 (7.3)
L-serine	3 (7.3)
Turmeric	3 (7.3)
Tauroursodeoxycholic acid	2 (4.9)
Magnesium	2 (4.9)
Nicotinamide riboside and pterostilbene	1 (2.4)
Creatine	1 (2.4)
Homeopathic remedies	1 (2.4)

^†^Each respondent could report more than one nutritional supplement; either by choosing predefined alternatives or give information as free text.

**Table 3:** RAND-12 component scores compared to the general population norm.

	ALS respondents, mean (SD)	General population norm, mean (SD)	Mean difference	95% confidence interval	*P* value (mean difference)^†^
PCS12	33.8 (8.03)	51.8 (9.48)	−15.1	−17.9, −12.3	<0.001
MCS12	39.7 (11.51)	51.8 (9.47)	−11.2	−15.1, −7.4	<0.001

^†^ *P* values were calculated using a paired sample *t*-test.

## Data Availability

The data used to support this study are available from the corresponding author upon request until 1st of may 2026.
